# Evolution by Any Other Name: Antibiotic Resistance and Avoidance of the E-Word

**DOI:** 10.1371/journal.pbio.0050030

**Published:** 2007-02-13

**Authors:** Janis Antonovics, Jessica L Abbate, Christi Howell Baker, Douglas Daley, Michael E Hood, Christina E Jenkins, Louise J Johnson, James J Murray, Vijay Panjeti, Volker H. W Rudolf, Dan Sloan, Joanna Vondrasek

## Abstract

The word "evolution" is rarely used in papers from medical journals describing antimicrobial resistance, which may directly impact public perception of the importance of evolutionary biology in our everyday lives.

The increase in resistance of human pathogens to antimicrobial agents is one of the best-documented examples of evolution in action at the present time, and because it has direct life-and-death consequences, it provides the strongest rationale for teaching evolutionary biology as a rigorous science in high school biology curricula, universities, and medical schools. In spite of the importance of antimicrobial resistance, we show that the actual word “evolution” is rarely used in the papers describing this research. Instead, antimicrobial resistance is said to “emerge,” “arise,” or “spread” rather than “evolve.” Moreover, we show that the failure to use the word “evolution” by the scientific community may have a direct impact on the public perception of the importance of evolutionary biology in our everyday lives.

To establish whether the word “evolution” is used with different frequency by evolutionary biologists versus researchers in the medical fields, we searched scientific journals published since 2000 for research papers and reviews dealing with antimicrobial resistance. To find these papers, we used standard search engines and databases to identify papers with “antimicrobial resistance” or “antibiotic resistance” (or with names of specific antibiotics) in the titles or abstract. We deliberately did not include the word “evolution” in the searches, so as not to bias our findings in favor of articles with this word. However, we chose for further analysis only those articles that were obviously describing the evolution of antimicrobial resistance, and excluded those that described, for example, the biochemical basis of resistance or the pharmacology of antimicrobial agents. The articles were chosen in an unbiased manner by several readers who each independently read the first papers they found that met these criteria. We compared 15 articles that were primarily published in evolutionary journals (such as *Evolution*, *Genetics*, and *Proceedings of the Royal Society of London Series B*) with 15 articles that were published in primarily medical journals (such as *The Lancet*, *The New England Journal of Medicine*, and *The Journal of Antimicrobial Chemotherapy*). (A list of the papers and articles that are the basis of the results reported here is available in Text S1.)

Each reader then read the articles in their entirety. In each paper we explicitly noted and counted the words or phrases (see below) that were used to describe the evolutionary process, in order to obtain the proportion of times that the actual word “evolution” (or its lexemes such as “evolutionary” or “evolving”) was used when reference was being made to the evolutionary process. Although we deliberately read equal numbers of articles in the two types of journals, we actually found that by far the majority of publications on the evolution of antibiotic resistance are in the medical field, and not in academic evolutionary biology or genetics journals. The evolution of antibiotic resistance, while critically important from a medical viewpoint, is no longer in and of itself a novel finding in evolutionary biology.

The results of our survey showed a huge disparity in word use between the evolutionary biology and biomedical research literature (Figure 1). In research reports in journals with primarily evolutionary or genetic content, the word “evolution” was used 65.8% of the time to describe evolutionary processes (range 10%–94%, mode 50%–60%, from a total of 632 phrases referring to evolution). However, in research reports in the biomedical literature, the word “evolution” was used only 2.7% of the time (range 0%–75%, mode 0%–10%, from a total of 292 phrases referring to evolution), a highly significant difference (chi-square, *p* < 0.001). Indeed, whereas all the articles in the evolutionary genetics journals used the word “evolution,” ten out of 15 of the articles in the biomedical literature failed to do so completely. Instead, 60.0% of the time antimicrobial resistance was described as “emerging,” “spreading,” or “increasing” (range 0%–86%, mode 30%–40%); in contrast, these words were used only 7.5% of the time in the evolutionary literature (range 0%–25%, mode 0%–10%). Other nontechnical words describing the evolutionary process included “develop,” “acquire,” “appear,” “trend,” “become common,” “improve,” and “arise.” Inclusion of technical words relating to evolution (e.g., “selection,” “differential fitness,” “genetic change,” or “adaptation”) did not substantially alter the picture: in evolutionary journals, evolution-related words were used 79.1% of the time that there was an opportunity to use them (range 26%–98%, mode 50%–60%), whereas in biomedical journals they were used only 17.8% of the time (range 0%–92%, mode 0%–10%).

**Figure 1:**
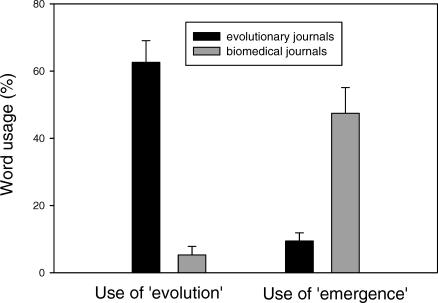
Frequency of Use of Words to Describe the Evolutionary Process in Evolutionary Journals versus Biomedical Journals The left-hand pair of bars show percentage use of the word “evolution,” and the right-hand pair of bars show percentage use of the words “emerge,” “arise,” or “increase.” Data shown are unweighted means and standard errors, based on 15 papers in evolution or genetics journals and 15 papers in biomedical journals.

In spite of the disparity in word use, we found that the papers in the medical literature generally included professional and competent descriptions of evolutionary processes. At times words such as “develop” or “acquire” did creep in, but egregiously misleading phrases were relatively rare. For example, once we found the wording “bacteria had learned to resist antibiotics” and at another time “the activity of antimicrobial agents had decreased” (which, if read literally, implies that the antimicrobials themselves were changing rather than that the pathogens were evolving). But these were exceptions.

In reading these papers, we found no evidence that deliberate efforts were being made by medical researchers to deny that evolutionary processes were involved in the increase of antibiotic resistance. The frequent use of the term “emergence” rather than “evolution” seemed more to be the result of a simplified phraseology that has “emerged and spread” out of habit and repeated usage. It may also be that many nonprofessional evolutionary biologists consider “evolution” to be a rather nonspecific word meaning “gradual change,” and that “emergence” more explicitly incorporates the component aspects of the evolutionary process, namely, mutation, recombination, and/or horizontal transfer of resistance. The word “spread” may, similarly, appear to incorporate the component processes of transmission, horizontal transfer, and increase in allele frequency. While these processes are recognized by professional evolutionary biologists as important aspects of evolutionary change, biomedical researchers may have the sense that the word “evolution” is itself too imprecise. Indeed, evolutionary biologists are sometimes accused of focusing too much attention on “change in gene frequency” rather than on the origin of variants by mutation and recombination, or on the consequences of changes in allele frequency for numerical abundance and distribution.

There is also the possibility that the failure to use the word “evolution” may reflect the mistaken sense that evolution implies processes that are long past, slow, and imperceptible. This is more worrying, as it fails to acknowledge the importance of evolution as a powerful force in present-day populations of all organisms, and not only microbes.

A critical question is whether avoidance of the word “evolution” has had an impact on the public perception of science. To investigate this, we examined whether the use of the term “evolution” in the scientific literature affects the use of this word in the popular press, i.e., whether there is evidence for “cultural inheritance” of word use. We searched articles on antimicrobial resistance in national media outlets, such as *The New York Times*, *The Washington Post*, *Fox News*, and the *BBC* (Text S1). Our results showed that the proportion of times the word “evolution” was used in a popular article was highly correlated with how often it was used in the original scientific paper to which the popular article referred (Figure 2). This clearly shows that the public is more likely to be exposed to the idea of evolution and its real-world consequences if the word “evolution” is also being used in the technical literature.

**Figure 2:**
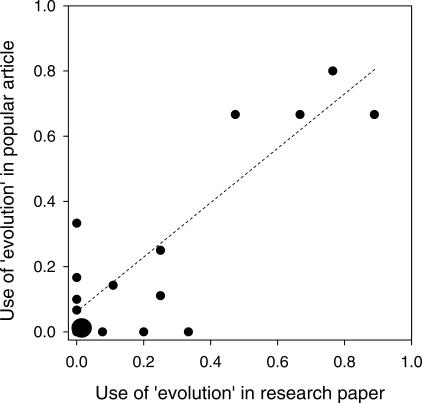
Use of “Evolution” in Popular Articles Based on Research Papers This graph shows the relationship between the frequency of use of the word “evolution” in popular press articles addressing antimicrobial resistance and the frequency of its use in the corresponding research article. Most of the articles included were in the biomedical literature (Text S1). The point at the origin represents nine pairs for which “evolution” was mentioned neither in the scientific nor in the popular version. The regression is highly significant (d.f. = 21, *p* < 0.0001, ß = 0.76; weighted arcsine square root transformed; points and fitted line in figure represent untransformed data).

We wondered whether these patterns were changing, so we carried out a survey of the use of the word “evolution” from 1991 to 2005 in the titles and abstracts of papers published in 14 scientific journals, as well as in the titles of proposals funded by both the US National Science Foundation (Division of Environmental Biology) and the US National Institutes of Health (National Institute of General Medical Sciences). The results showed that the use of the word “evolution” was actually increasing in all fields of biology, with the greatest relative increases in the areas of general science and medicine (Figure 3). This reflects the growing importance of evolutionary concepts in the biomedical field, and highlights even more the strange rarity with which the word “evolution” is used in the biomedical literature dealing with antimicrobial resistance. It has been repeatedly rumored (and reiterated by one of the reviewers of this article) that both the National Institutes of Health and the National Science Foundation have in the past actively discouraged the use of the word “evolution” in titles or abstracts of proposals so as to avoid controversy. Indeed, we were told by one researcher that in the title of one proposal, the authors were urged to change the phrase “the evolution of sex” to the more arcanely eloquent wording “the advantage of bi-parental genomic recombination.”

**Figure 3:**
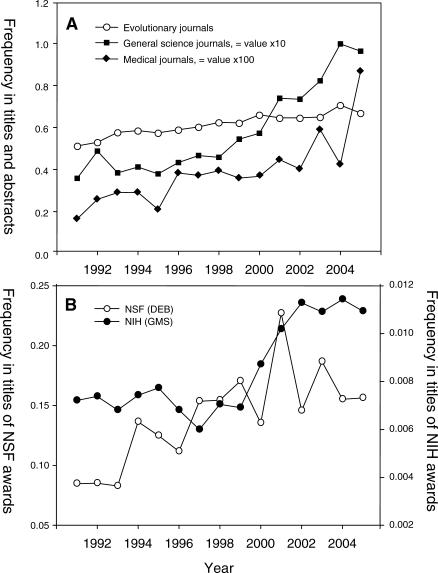
Change over Time in the Frequency of Use of the Word “Evolution” in Journals and Grant Proposals This figure shows change in the frequency of use of the word “evolution” in (A) paper titles and abstracts for journals classified by type and (B) titles of funded research proposals classified by US federal granting agency. Note that the data for general science journals and medical journals are shown at 10 and 100 times their values, respectively. Analysis of covariance (log of arcsine square root transformed data) showed that the rate of increase of use of the word “evolution” was significantly greater in the journal categories of general science and medical than in the evolutionary category (*p* < 0.002). Journal classification was as follows: evolutionary journals: *Evolution*, *Genetics*, *Heredity*, *Journal of Evolutionary Biology*, *Journal of Molecular Evolution*, *Molecular Biology and Evolution*; general science journals: *Nature*, *Nature Genetics*, and *Science*; medical journals: *BMJ*, *Clinical Infectious Diseases*, *JAMA*, *The Lancet*, and *The New England Journal of Medicine*. Funding data are from the online data retrieval systems of the National Science Foundation (Division of Environmental Biology) (NSF [DEB]) and National Institutes of Health (National Institute of General Medical Sciences) (NIH [GMS]).

Nowadays, medical researchers are increasingly realizing that evolutionary processes are involved in immediate threats associated with not only antibiotic resistance but also emerging diseases [1,2]. The evolution of antimicrobial resistance has resulted in 2- to 3-fold increases in mortality of hospitalized patients, has increased the length of hospital stays, and has dramatically increased the costs of treatment [3,4]. It is doubtful that the theory of gravity (a force that can neither be seen nor touched, and for which physicists have no agreed upon explanation) would be so readily accepted by the public were it not for the fact that ignoring it can have lethal results. This brief survey shows that by explicitly using evolutionary terminology, biomedical researchers could greatly help convey to the layperson that evolution is not a topic to be innocuously relegated to the armchair confines of political or religious debate. Like gravity, evolution is an everyday process that directly impacts our health and well-being, and promoting rather than obscuring this fact should be an essential activity of all researchers.

## Supporting Information

Text S1 Accessory Materials (125 KB DOC).
