# Supplementary material for: Evolution by Any Other Name: Antibiotic Resistance and Avoidance of the E-Word
Source: PLoS Biol. 2007 Feb 13;5(2):e30. doi: 10.1371/journal.pbio.0050030 (PMC1796926; doi:10.1371/journal.pbio.0050030)
Supplement: Text S1 — (125 KB DOC). [file pbio.0050030.sd001.doc]

**Accessory Materials 1**

**References used for word counts**

**Articles in Evolutionary Journals**

Anderson JB, Sirjusingh C, Parsons AB, et al. 2003. Mode of selection and experimental evolution of antifungal drug resistance in *Saccharomyces cerevisiae*. *Genetics* 163: 1287-1298.

Barlow M, Hall BG. 2003. Experimental prediciton of the natural evolution of antibiotic resistance. *Genetics* 163: 1237-1241.

Bensasson D, Boore JL, Niesen, KM. 2004. Genes without frontiers? *Heredity* 92: 483-489.

Boni, MF, Feldman MW. 2005. Evolution of antibiotic resistance by human and bacterial niche construction. *Evolution* 59: 477-491

Dahlberg C, Chao L. 2003. Amelioration of the cost of conjugative plasmid carriage in *Escherichia coli* K12  *Genetics* 165: 1641-1649.

Denamur E, Tenaillon O, Deschamps C, et al. 2005. Intermediate mutation frequencies favor evolution of mulit-drug resistance in *Escherichia coli*. *Genetics* 171: 825-827.

Dugatkin LA, Perlin M, Lucas JS, Atlas R. 2005. Group-beneficial traits, frequency-dependent selection and genotypic diversity: an antibiotic resistance paradigm. *Proc. Roy. Soc. Lond. B.* 272: 79-83.

Iwasa Y, Michor F, Nowak MA. 2003. Evolutionary dynamics of escape from biomedical intervention. *Proc. Roy. Soc. Lond. B* 270: 2573–2578.

Levin BR, Perrot V, Walker N. 2000. Compensatory mutations, antibiotic resistance and the population genetics of adaptive evolution in bacteria. *Genetics* 154: 985-997.

Nandi S, Maurer JJ, Hofacre C, Summers AO. 2004. Gram-positive bacteria are a major reservoir of Class 1 antibiotic resistance integrons in poultry litter. *PNAS*. 18: 7118-7122.

Perron GG, Zasloff M, Bell G. 2006. Experimental evolution of resistance to an antimicrobial peptide. *Proc. Roy. Soc. Lond. B* 273: 251-256.

Reynolds MG. 2000. Compensatory evolution in rifampin-resistant *Escherichia coli*.*Genetics* 156: 1471-1481.

Rokas A, Holland PWH. 2000. Rare genomic changes as a tool for phylogenetics. *Trends in Ecology and Evolution* 15: 454-459.

Salipante SJ, Hall BG. 2003. Determining the limits of the evolutionary potential of an antibiotic resistance gene. *Mol. Biol. Evol.* 20: 653-659.

Walker ES, Levy F. 2001. Genetic trends in a population evolving antibiotic resistance. *Evolution* 55: 1110-1122.

**Articles in medical journals**

Bright RA, Shay DK, Shu B, et al. 2006. Adamantine resistance among influenza A viruses isolated early during the 2005-2006 influenza season in the United States.  *JAMA*. Published online Feb 2, 2006.

Enne VI, Livermore DM, Stephens P, et al. 2001. Persistence of sulphonamide resistance in *Escherichia coli* in the UK despite national prescribing restriction. *The Lancet* 357: 1325-1328.

Foster KR, Grundman H. 2006. Do we need to put society first? The potential for tragedy in antimicrobial resistance. *PLoS Medicine* 3(2): e29.

Gillespie SH. 2002. Evolution of drug resistance in *Mycobacterium tuberculosis*: clinical and molecular perspective. *Antimicrobial Agents and Chemotherapy* 46: 267-274

Goossens H, Ferech M, Stichele RV, et al. 2005. Outpatient antibiotic use in Europe and association with resistance: a cross-national database study. *Lancet* 365: 579-587

Levy SB, Marshall B. 2004. Antibacterial resistance worldwide: causes, challenges and responses*. Nature Medicine* 10: S122-S129.

Lukehart SA, Godornes C, Molini BJ, et al. 2004. Macrolide resistance in *Treponema pallidum* in the United States and Ireland. *New England Journal of Medicine* 351: 154-158.

Man P de, Verhoeven BAN, Verbrugh HA, et al. 2000. An antibiotic policy to prevent emergence of resistant bacilli. *The Lancet* 355: 973-978.

McCormick AW, Whitney CG, Farley MM, et al. 2002 Geographic diversity and temporal trends of antimicrobial resistance in *Streptococcus pneumoniae* in the United States. *Nature Medicine* 8: 369-1375.

Neuhauser MM, Weinstein RA, Rydman R, et al. 2006. Antibiotic resistance among gram-negative bacilli in US intensive care units. Implications for fluoroquinolone use. *Journal of the American Medical Association* 289: 885-888.

Okeke IN, Laxminarayan R, Bhutto ZA, et al. 2005. Antimicrobial resistance in developing countries. Part I: Recent trends and current status. *The Lancet Infectious Diseases* 5: 481-493

Oteo J, Campos J, Baquero F, et al. 2002. Antibiotic resistance in 1962 invasive isolates of *Escherichia coli* in 27 Spanish hospitals participating in the European Antimicrobial

Resistance Surveillance System (2001). *Journal of Antimicrobial Chemotherapy* 50: 945-952.

Rychlik, Gregorova, Hradecka. 2006. Distribution and function of plasmids in *Salmonella enterica*. *Veterinary Microbiology* 112: 1-10.

Stewart PS, Costerton JW. 2001. Antibiotic resistance of bacteria in biofilms. *The Lancet* 358: 135-138

White DG, Zhao S, Sudler R, et al. 2001. The isolation of antibiotic-resistant *Salmonella* from retail ground meats. *New England Journal of Medicine* 345: 1147-1154.

**Accessory Materials 2**

References used for comparison of word-use in popular and scientific articles

| parent | 0 | 5 | Tsiodras S, Gold HS, Sakoulas G, et al. 2001. Linezolid resistance in a clinical isolate of *Staphylococcus aureus*. *The Lancet* 358: 207-208. |
| --- | --- | --- | --- |
| offspring1 | 0 | 3 | Branswell H. 2001. Superbugs beat another drug: antibiotic on market for one year already facing staph resistance. *The Gazette*, Montreal, Quebec, July 20. |
| offspring2 | 0 | 5 | Branswell H. 2001. MDs concerned drug succumbed so quickly. *Toronto Star*, Friday Edition, July 20. |
|  |  |  |  |
| parent | 2 | 6 | Kuroda M, Ohta T, Uchiyama I, et al. 2001. Whole genome sequencing of meticillin-resistant *Staphylococcus aureus*. *The Lancet* 357: 1225-1240 |
| offspring | 0 | 2 | Derbyshire D. 2001. MDs concerned drug succumbed so quickly. *Toronto Star*, Friday Edition, July 20. |
|  |  |  |  |
| parent1 | 0 | 8 | White DG, Zhao S, Sudler R. et al. 2001. The isolation of antibiotic-resistant *Salmonella* from retail ground meats. *New England Journal of Medicine* 345:1147-1154. |
| parent2 | 0 | 9 | McDonald LC, Rossiter S, Mackinson C., et al. Quinupristin-dalfopristin-resistant *Enterococcus faecium* on chicken and in human stool specimens. *New England Journal of Medicine* 345:1155-1160. |
| parent3 | 0 | 0 | Sorensen TL, Blom M, Monnet DL, et al. 2001. Transient intestinal carriage after ingestion of antibiotic-resistant *Enterococcus faecium* from chicken and pork.  *New England Journal of Medicine* 345:1161-1155. |
| offspring 1 | 0 | 5 | Brody JE. 2001. Studies find resistant bacteria in meats. *New York Times*, October 18. |
| offspring 2 | 0 | 5 | Fauber, J. 2001. The perils of animal antibiotics; Widespread use of drugs on farm herds, flocks is raising concerns about creation of super microbes. *Milwaukee Journal Sentinel. News*, Pg. 01A. November 4, 2001. |
|  |  |  |  |
| parent | 0 | 8 | Gonzales RD, Schreckenberger PC, Graham MB, et al. 2001. Infections due to vancomycin-resistant *Enterococcus faecium* resistant to Linezolid. *The Lancet* 357:1179. |
| offspring1 | 0 | 3 | Jamieson A. 2001. Superbugs fight back against drug. *The Scotsman*, April 13. |
| offspring2 | 1 | 7 | Derbyshire D. 2001. New drug is losing fight against superbug. *The Daily Telegraph* April 13. |
|  |  |  |  |
| parent | 0 | 11.5 | Whitney CG, Farley MM, Hadler J, et al. 2000. Increasing prevalence of multi-drug resistant *Streptococcus pneumoniae* in the United States. *New England Journal of Medicine* 343:1917-1924. |
| offspring | 0 | 5 | Gigliotta G. 2000. Key bacteria strain's drug resistance grows. *The Washington Post* December 28. |
|  |  |  |  |
| parent | 13 | 17 | Weinreich DM, Delaney NF, DePristo MA, et al. 2006. Darwinian evolution can follow only very few mutational paths to fitter proteins. *Science* 312:111-114. |
| offspring | 4 | 5 | Anonymous. 2006. Antibiotic resistance evolution is studied. *UPI NewsTrack*, April 11. |
|  |  |  |  |
| parent | 0 | 4 | Lukehart SA, Godornes C, Molini BJ, et al. Macrolide resistance in *Treponema pallidum* in the United States and Ireland. *New England Journal of Medicine* 351: 154-158. |
| offspring | 0 | 1 | Worcester S. 2004. Syphilis resistance. *Internal Medicine News,* August 15. |
| offspring | 0 | 4 | Paulson T. 2004. Antibiotic loses its effectiveness against syphilis; Scientists advise to stop treatment until further study. *Seattle Post-Intelligencer*, July 25. |
|  |  |  |  |
| parent | 2 | 8 | D'Costa VM, McGrann KM, Hughes D, et al. 2006. Sampling the antibiotic resistome. *Science* 311: 374-377. |
| offspring | 1 | 9 | Anonymous. 2006. Drug resistance: researchers use dirt to stay one step ahead of antibiotic resistance. *Obesity, Fitness and Wellness Week*, February 18, 2006. |
|  |  |  |  |
| parent | 0 | 2 | Fridkin SK et al. 2005. Methicillin-resistant *Staphylococcus aureus* disease in three communities. *New England Journal of Medicine* 352: 1436-1444. |
| offspring 1 | 1 | 5 | Barrow, K. 2005. Attack of the Superbugs: The Spread of Antibiotic-resistant Bacteria. May 18. *www.abcnews.com* |
| offspring 2 | 0 | 10 | Chong JR. 2006. Infection is Growing in scope, resistance. *Los Angeles Times*. Feb. 26. |
|  |  |  |  |
| parent | 0 | 5 | McDonald LC, Killgore GE, Thompson A, et al. 2005. An epidemic, toxin gene-variant strain of *Clostridium difficile*. *New England Journal of Medicine* 353:2433-2441 |
| offspring | 0 | 4 | Anonymous. 2005. Deadly bacterial illness linked to antibiotics spreading. *Associated Press*, December 2. *www.foxnews.com* |
|  |  |  |  |
| parent | 0 | 7 | Hay AD, Thomas M, Montgomery A, et al. 2005. The relationship between primary care antibiotic prescribing and bacterial resistance in adults in the community: a controlled observational study using individual patient data. *Journal of Antimicrobial Chemotherapy* 56:146-153 |
| offspring | 0 | 3 | Anonymous. 2005. *New York Times*, Aug. 30. |
|  |  |  |  |
| parent | 2 | 3 | Schuch R, Nelson D, Fischetti VA 2002. A bacteriolytic agent that detects and kills *Bacillus anthracis.*  *Nature* 418: 884-888 |
| offspring | 2 | 3 | Anonymous. 2002. *New York Times*, Aug 22. |
|  |  |  |  |
| parent | 1 | 13 | Howe RA, Monk A, Wootton M, et al. 2004. Vancomycin susceptibility within methicillin-resistant *Staphylococcus aureus* lineages. *Emerging Infectious Diseases* 10:855-857. |
| offspring | 0 | 8 | Anonymous. 2004. Hospital superbug deaths 'could double'. *Daily Mail* (UK), June 18. |
|  |  |  |  |
| parent | 0 | 1 | Elaine L. Larson, Susan X. Lin, Cabilia Gomez-Pichardo, and Phyllis Della-Latta, (2004) Effect of antibacterial home cleaning and handwashing products on infectious disease symptoms. A randomized, double-blind trial. *Annals of Internal Medicine*. 140:321-329 |
| offspring | 1 | 3 | Milloy S. 2004. Antibacterial reports cause public health scare. *Junk Science, Fox News*, March 5. www.foxnews.com. |
|  |  |  |  |
| parent | 2 | 10 | Neuhauser MM, Weinstein RA, Rydman R, et al. 2003. Antibiotic resistance among gram-negative bacilli in US intensive care units. Implications for fluoroquinolone use. *JAMA*.289:885-888. |
| offspring | 0 | 7 | Anonymous. 2003. New resistance to anthrax treatment. *Associated Press,* Feb 18. www.cbsnews.com. |
|  |  |  |  |
| parent | 2 | 8 | D’Costa VM, McGrann KM, Hughes DW, et al. 2006. Sampling the antibiotic resistome. *Science* 311:374-377. |
| offspring | 1 | 4 | Anonymous. 2006. Antibiotic resistance widespread in nature. *Forbes.com, Health*, January 19. |
|  |  |  |  |
| parent | 5 | 46 | Verhoef J, Fluit A. 2006. Surveillance uncovers the smoking gun for resistance emergence.  *Biochemical Pharmacology* 71: 1036-1041. |
| offspring | 1 | 7 | Anonymous. 2006. Drug resistance: surveillance uncovers smoking gun for antibiotic resistance emergence. *Biotech Week*, May 29. |
|  |  |  |  |
| parent | 0 | 11 | Chee-Sanford JC, Aminov RL, Krapac IJ, et al. 2001. Occurrence and diversity of tetracycline resistance genes in lagoons and groundwater underlying two swine production facilities. *Applied and Environmental Microbiology* 67: 1494-1502. |
| offspring | 1 | 6 | Spears T.  2001.'Superbugs' are escaping from the farm: From manure to farmers' fields and on into the environment. *The Ottawa Citizen*, June 4. |
|  |  |  |  |
| parent | 40 | 45 | Barlow M, Hall BG. 2002. Predicting evolutionary potential: in vitro evolution accurately reproduces natural evolution of the TEM beta-lactamase. *Genetics* 160: 823-832. |
| offspring | 4 | 6 | Chang A. 2002. Study finds laboratory evolution mimics natural evolution. *Associated Press State and Local Wire*. March 19. |
|  |  |  |  |
| parent | 9 | 19 | Holden MTG, Feil EJ, Lindsay JA, et al. 2004. Complete genomes of two clinical *Staphyococcus aureus* strains: evidence for rapid evolution of virulence and drug resistance. *Proceedings of the National Academy of Sciences* 101: 9786-9791. |
| offspring | 4 | 6 | Highfield, R. 2005. Superbug related to a bacteria carreid by third of population. *The Daily Telegraph,* February 25. |
|  |  |  |  |
| parent | 0 | 2 | Gilbert M, MacDonald J, Gregson D, et al.  2006. Outbreak in Alberta of community-acquired (USA300) with a history of drug use, homelessness or incarceration.  *Canadian Medical Association Journal* 175(2):149-154. |
| offspring | 0 | 8 | Branswell H.  2006.  Dangerous superbug taking off in Canada, moving beyond high-risk groups.  *CBC News Online*, June 27. |
|  |  |  |  |
| parent | 0 | 7 | Donovan DM, Dong S, Wes G, et al. 2006. Peptidoglycan hydrlase fusions maintain their parental specificities.  *Applied and Environmental Microbiology* 72: 2988-2996. |
| offspring | 0 | 1 | Anonymous. 2006. Antimicrobials fight mastitis-causing bacteria. *Capital Press Agriculture Weekly*, Jul 14. |
|  |  |  |  |
| parent | 0 | 1 | Sebaihia M, Wren BW, Mullany P, et al.  2006.  The multidrug-resistant human pathogen *Clostridium difficile* has a highly mobile, mosaic genome. *Nature Genetics* 38:779-786. |
| offspring | 0 | 3 | Anonymous 2006 How hospital bug can evade attack.  *BBC News*, Jun 25. |
